# High Temperature Rotational Rheology of the Seed Flour to Predict the Texture of Canned Red Kidney Beans (*Phaseolus vulgaris*)

**DOI:** 10.3390/foods9081002

**Published:** 2020-07-26

**Authors:** Richard Park, Laura Roman, Louis Falardeau, Lionel Albino, Iris Joye, Mario M. Martinez

**Affiliations:** 1 School of Engineering, University of Guelph, Guelph, ON N1G 2W1, Canada; parkr@uoguelph.ca; 2 Bonduelle Americas, 540 Chemin des Patriotes, St-Denis-Sur_Richelieu, QC J0H 1K0, Canada; louis.falardeau@bonduelle.com; 3 Bonduelle, Rue Nicolas Appert, F-59653 Villeneuve d’Ascq, France; lionel.albino@bonduelle.com; 4 Department of Food Science, University of Guelph, Guelph, ON N1G 2W1, Canada; ijoye@uoguelph.ca; 5 Department of Food Science, iFOOD Interdisciplinary Center, Aarhus University, 8200 Aarhus N, Denmark

**Keywords:** rheology, pasting profile, starch gelatinization, red kidney beans, canning

## Abstract

The pasting profile of starchy tissues is conventionally measured by recording the apparent viscosity (η) in heating/cooling cycles. However, conventional rheometers show critical limitations when the starch is embedded in compact protein-rich cotyledon matrices, as occurs in pulses. In this work, the pasting profile of 13 red kidney beans (*Phaseolus vulgaris*) from the same cultivar but different growing locations was investigated using a heating/cooling cycle at higher temperature (130 °C) and pressurized conditions, using both water and brine as cooking solvents. It was hypothesized that the continuous measure of η at these conditions of flours from the dry seed would correlate with the texture, as determined by the mini-Kramer cell, of the beans after the entire process of soaking and canning. Furthermore, mechanistic answers were obtained by investigating their composition (starch, protein, and ash content) and physical properties (water holding capacity, seed ratio and weight). Interestingly, as opposed to the pasting profile at 95 °C, pasting indicators at 130 °C, including trough and final viscosity, strongly correlated with starch and protein content, seed coat ratio and, remarkably, with the firmness of the beans after canning when brine was incorporated. These results clearly show that small beans with a high protein content would bring about a more compact matrix that restricts starch from swelling and results in canned beans with a hard texture, which can be predicted by a lower pasting profile of the whole bean flour.

## 1. Introduction

The demand for plant-based proteins is rapidly growing with increased awareness of the carbon footprint caused by a meat-based diet. Among plant protein sources, pulses are in the spotlight for their high content in protein, carbohydrates, including dietary fiber, and minerals and vitamins [1,2,3]. In fact, the consumption of pulses has been associated with a reduced prevalence of type 2 diabetes and cardiovascular disease [4]. Abdullah et al. [4] estimated that an increased consumption of pulses to 100 g/day per person could save up to CAD USD 315 million on healthcare costs annually in Canada, attributed to anticipated reduced incidence of type 2 diabetes, colon cancer, and cardiovascular disease. Although the consumption of pulses is on the rise, already reaching 10–40 kg/capita per year in Latin America and South Asia, its consumption remains lower than health recommendations in some other countries. As an example, pulse consumption still remains low in Canada, with only 13% of the adult population reporting the consumption of pulses (at an average of 113 g) on any given day [5]. 

Traditional cooking and commercial canning processes are used to soften the bean texture, which results in a safe and palatable food. However, traditional cooking is labor- and time-intensive, and often unsuitable for urban settings where preparation time is a major constraint. Thus, ready-to-eat canned beans play a major role in some individuals’ diet and are in high demand by the food industry, fast-food restaurants and households. The quality of canned beans can vary greatly, and consumers demand beans with proper integrity, a pleasant flavor profile, and pleasant firmness/tenderness [6]. The firmness of canned beans, one of their most important quality traits, varies from too firm (‘tough beans’) to too soft (‘mushy beans’), with texture standards established for each type and pack style of canned beans [7]. Instrumental texture readings determine the firmness or softness of a sample and serve as a proxy of how beans will feel in the mouth, i.e., they serve as a useful estimator for consumer’s acceptance. In addition, previous studies identified a positive correlation between appearance and texture [8], since excessive tissue softening might often be accompanied by a loss of whole bean integrity [9,10,11]. The texture of canned beans is influenced by a plethora of intrinsic (microstructure and composition as affected by genotype and growing conditions) and extrinsic factors (seed handling during storage and processing conditions during soaking, blanching and canning) and it is assessed by determining the amount of force (kg) required to crush a sample of 100 g of cooked beans [10,11]. However, this method requires beans going through the entire time-consuming canning process prior to texture evaluation and the use of a significant amount of dry beans. Thus, rapid methods for predicting the texture of canned beans could become instrumental for both bean breeders, producers and processors. Mendoza et al. [11] investigated the feasibility of using non-destructive optical sensors (visible/near-infrared spectroscopy and hyperspectral imaging) as a predictor for canned black bean firmness. After building a model using parameters from spectroscopy and hyperspectral imaging of dry beans against actual obtained canned bean firmness values, the calibration and prediction data overlapped well with the prediction models, having a regression line of R = 0.886 for visible/near-infrared spectroscopy and R = 0.844 for hyperspectral imaging. However, large datasets using various phenotypic diversities must be obtained to be able to develop a robust prediction model with other bean varieties. Moreover, data must be preprocessed using different smoothing and deconvolution methods. Overall, this might require the presence of skillful workers and a significant amount of time. 

The structure of the dry bean comprises a seed coat and an embryonic cotyledon. The cotyledon makes up approximately 90% of the total dry weight in the mature bean with around 40% starch, 27.5% protein, 3.5% ash and 1.65% lipids. During processing, protein will absorb water at room temperature during soaking, which will subsequently enable the starch (surrounded by protein bodies) to absorb water during the hydrothermal processing, such as blanching and cooking [12,13]. Khanal et al. [10] found that the genotype and location of harvest both had a significant impact on the protein and starch content of the beans, as well as on the texture of the beans after canning. Hence, based on the water (or brine) absorption and swelling capacity of the most predominant cotyledon components, it is hypothesized that rheological indicators from a dispersion of dry bean flour in water would correlate with the final texture of canned whole beans. 

The apparent viscosity measured during a heating/cooling cycle, i.e., pasting profile, could provide useful indicators of the behavior of samples during hydrothermal processing. Typically, the maximum hold temperature during this rheological test is 95 °C, which is the maximum temperature attainable by most instruments without water evaporation. Briefly, the apparent viscosity of starch/flour in a liquid medium starts increasing rapidly mostly due to the swelling of starch granules (starting with the disruption of the hydrogen bonds in amorphous regions and water absorption). As the temperature increases, more hydration and more swelling occurs in amorphous regions, pulling apart crystallites, whose regions also eventually undergo hydration and melt [14]. Leaching of polymer molecules, such as soluble pectin, amylopectin and especially amylose, also occurs. Eventually, a peak viscosity, primarily resulting from swollen granules, is reached. During the 95 °C hold, the fragile swollen flour particles disintegrate under the shear conditions of the instrument, and the viscosity decreases to a trough viscosity (a process called breakdown). The degree of fragmentation depends on the shear time, and nature of the sample. As the hot paste begins to cool, it develops distinct solid properties and the apparent viscosity raises again, i.e., gelation occurs [14]. These rheological measurements can be obtained with the so-called empirical rheometers that are robust, capable of withstanding demanding factory environments and do not require highly skilled or technically trained personnel. Interestingly, some of these apparatuses, such as the RVA 4800, are able to reach temperatures of up to 140 °C under pressurized conditions to resemble hydrothermal processing above 95 °C (typical upper limit of commercial viscosimeters due to water evaporation), which is particularly important when working with protein-rich tissue matrices that make starch more resistant to gelatinization [15]. Thus, the main aim of this work is to find meaningful correlations between the texture of canned beans and rheological indicators of the dry seed flour during a heating cooling cycle using an RVA 4800, which could result in significant savings of time during the textural evaluation of canned beans. Furthermore, physical and compositional analyses, including weight per bean, seed coat (hull) to whole-seed ratio, water-holding capacity, protein, starch and ash content, were measured to provide mechanistic understanding about the factors affecting canned bean texture, which has never been provided in a systematic manner.

## 2. Materials and Methods

### 2.1. Materials

Thirteen whole red kidney beans (*Phaseolus vulgaris*), from the same variety but varying in growing locations, were provided by Bonduelle Group in collaboration with Hensall Co-op. To obtain whole bean flours for analyses, beans were cryo-milled for 2 min in a coffee grinder (Black+Decker CBG100SC, Middleton, WI, USA) and sifted under a 250-micrometer mesh sieve (Retsch AS 200, Haan, Germany). The ground whole flours were stored in a freezer at −20 °C until further analyses in order to minimize any enzymatic activity in the sample. For individual compositional analyses on seed coat and cotyledon of the beans, coats and cotyledons were manually separated, cryo-milled and stored as aforementioned. Cotyledons were cryo-milled as above detailed for the whole beans, but for the seed coat, a ball mill (Retsch PM 100, Haan, Germany) set at 6 min at 600 rpm was used, as the coats were too light and difficult to mill otherwise.

### 2.2. Methods

#### 2.2.1. Compositional Analysis of Beans

Proximate compositional analyses were performed at least in duplicate. Moisture content of whole bean flours was measured using an automated moisture analyzer (Sartorius MA35, Goettingen, Germany) in accordance with AACC method 44-15.02 [16]. Ash in the whole flours was analyzed according to AACC 923.03 method. Protein content of seed coat and cotyledon was determined using the combustion method according to AACC method 46-30.01 with an automated Dumas protein analysis system [16] (Leco FP-528, St. Joseph, MI, USA), using the factor of 6.25 to convert the measured nitrogen into protein. Total starch content of cotyledon was measured using the Megazyme total starch kit (K-TSTA-100A, Wicklow, Ireland) according to AACC method 76-13. [16]. 

#### 2.2.2. Physical Analysis of Beans

Seed weight was determined in duplicate by weighing 50.0 ± 0.2 g of beans and dividing it by the number of beans manually counted in the 50 g of sample. The water-holding capacity (WHC) of intact whole beans was measured in accordance to the AACC 56-35 method [16] with some modifications. A total of 25 g of beans were soaked in 100 g of distilled water at 1:4 (sample:water, *w*/*w*) ratio for 16 h at room temperature. After draining, the beans were left to dry on a paper towel for 5 min and weighed. WHC was assessed in duplicate and was calculated on dry basis as follows:

(1) $$\mathrm{WHC}\;\left(\%\right)=\frac{100\times \left(W_{2}-W_{1}\right)\;}{W_{1}\times \left(100-M_{c}\right)}\times 100\;$$

where W_1_ = weight of seeds before soaking, W_2_ = weight of seeds after soaking, and M_c_ = the moisture content, expressed as a percentage by mass, of the seeds. 

Seed coat to whole seed ratio was determined in triplicate by weighing and soaking 25 g of beans at room temperature in distilled water for 75 min to facilitate separation of the coat layer from the cotyledon tissue. After 75 min, coats were separated manually from cotyledons and the two fractions were dried in an oven for 16 h at 40 °C until constant weight. After 16 h, seed coat and cotyledon fractions were weighed separately and seed coat to whole seed ratio was calculated as shown in Equation (2).

(2) $$\mathrm{Seed}\;\mathrm{coat}\;\mathrm{to}\;\mathrm{whole}\;\mathrm{seed}\;\mathrm{ratio}=\frac{\mathrm{Weight}\;\left(g\right)\;\mathrm{of}\;\mathrm{dried}\;\mathrm{seed}\;\mathrm{coat}}{\left(\mathrm{Weight}\;\left(g\right)\;\mathrm{of}\;\mathrm{dried}\;\cot\mathrm{yledon}+\mathrm{dried}\;\mathrm{seed}\;\mathrm{coat}\right)}\times 100$$

#### 2.2.3. Pasting Profiles of Whole Bean Flours

**Figure 1:**
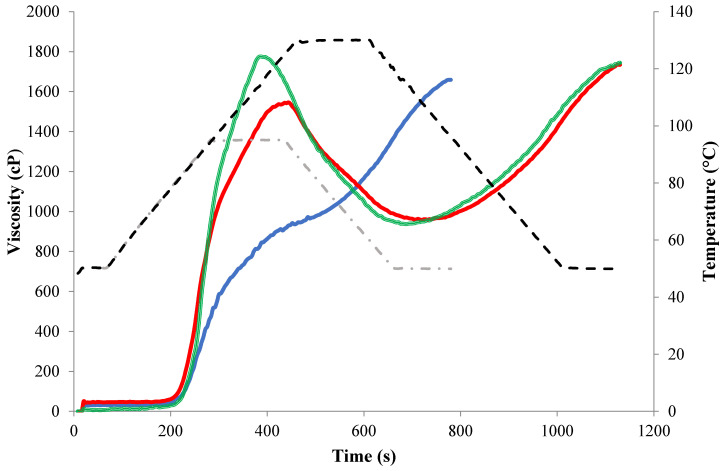
Pasting profiles of whole bean flour with different mediums and two different temperature profiles. The same red kidney bean flour was used to perform the three pasting profiles represented in this figure. Blue line, pasting profile with holding time at 95 °C using water; Red line, pasting profile with holding time at 130 °C using water; Green line, pasting profile with holding time at 130 °C using brine (1.5% sucrose, 1.2% NaCl, 0.03% CaCl_2_). Discontinues grey and black lines represent the temperature as a function of time in standard (95 °C) and pressurized high temperature (130 °C) RVA tests, respectively.

The pasting profiles of whole bean flours were measured using a Rapid Visco-Analyzer (RVA 4800 Perten Instruments, Sydney, Australia) equipped with a canister and paddle system able to work under retort (high pressure and high temperature) conditions. Pasting profiles of the flours were obtained following the typical standard pasting cycle at 95 °C hold temperature according to AACC method 61-02.01 [16], as well as the high-temperature pasting cycle (130 °C hold temperature), as described by Perten Instruments [17] using 4.0 g of flour and 25 mL ultrapure water (Table 1). The 130 °C hold pasting profiles were also obtained using brine (1.5% sucrose, 1.2% NaCl, 0.03% CaCl_2_) instead of water, with a pH of 5.9. The brine was prepared as reported by Mendoza et al. [11]. A comparative example of RVA profiles at 95 and 130 °C from the same red kidney bean flour sample are shown in Figure 1, where a noticeable underdeveloped pasting profile at 95 °C is depicted. The pasting profiles were used to obtain the following parameters based on the viscosity values: peak viscosity (PV, highest viscosity reached during heating), trough viscosity (lowest viscosity reached during the holding/cooling stage), breakdown viscosity (difference between the peak viscosity and the trough), final viscosity (viscosity at the end of the test) and setback (difference between trough viscosity and final viscosity). The time to reach peak viscosity (peak time) and the time that gives rise to 50% of peak viscosity (time for PV/2) were also included as descriptors of the resistivity of the flour particles to swell during heating. Finally, the temperature at which the viscosity starts to increase rapidly is also recorded (pasting temperature). RVA measurements were performed at least in duplicate. It is noteworthy that the relative standard deviation, RSD = (SD/average) × 100, was <10% for 203 out of the 208 values obtained running the RVA, and, in most cases, <5%.

#### 2.2.4. Canning of Whole Beans

For canning, 50.0 ± 0.2 g of raw beans were weighed, counted and soaked for 6 h in four times the seed weight of distilled water (~200 g). After soaking, the beans were drained and allowed to dry on a paper towel for 5 min. Subsequently, the soaked beans were placed in 250 mL mason jars, covered with brine (1.5% sucrose, 1.2% NaCl, 0.03% CaCl_2_) up to 2 cm from the top of the jar (120% of soaked weight), as reported in Mendoza et al. [11]. Using an automated pressure cooker filled with 1.5 L of ultrapure water (Instant Pot Max, Kanata, ON, Canada), the samples were pre-heated to 121 °C and 15 psi for 24 min. Afterwards, the jars were retorted at 121 °C, 15 psi for 6 min (according to the canning settings) and then the steam was immediately released (quick release setting). The number of jars in the cooker was controlled to seven at a time, with one of the jars being filled with distilled water and placed in the middle of the cooker, to make sure all the six remaining jars containing beans received the same amount of heat. After cooking, the jars were cooled under tap water (20 °C) for 20 min, and stored at room temperature until further analysis. This cooling was performed to reduce integrity loss from overcooking and minimize growth of thermophilic bacteria [18]. Cooking experiments were performed in duplicate.

#### 2.2.5. Texture Analysis of Canned Beans

The canned beans were analyzed 24 h after retorting. Jars were opened and beans were drained and dried on a paper towel for 5 min. Firmness analysis was conducted using a texture analyzer (TA.XT2. plus, Texture Technologies. Corp., Scarsdale, NY, USA) equipped with a 20 kg load cell according to the AACC method 56-36.01 [19]. 7.5 ± 0.5 g of beans were loaded into a mini Kramer shear cell holder (HDP/MK05, Stable Micro Systems, Surrey, UK). The probe started at 30 mm above zero, and cooked beans were compressed and extruded at 1.5 mm/s with a five-blade shear probe until reaching the cell’s bottom. The firmness (N) of the sample was determined as the maximum force of the peak obtained. An average of minimum four replications was used.

#### 2.2.6. Statistical Analysis

Statistical analysis was conducted using Statistical Package for the Social Sciences (SPSS), including the *t*-test for significance of differences in averages between the two RVA mediums, significance of difference between samples using analysis of variance (one way ANOVA—post-hoc with Tukey’s HSD), Pearson’s bivariate correlations matrix, and linear regression—the best model after correlation. All tests were done at a 95% confidence interval.

## 3. Results

### 3.1. Composition, Water Holding Capacity and Physical Properties of Dry Beans

Results for compositional analyses of red kidney beans are reported in Table 2. The moisture of the whole seeds ranged from 12.64 to 16.86% (always below 17%). During harvest and upon arrival of the beans to the reception facility, beans are usually subjected to drying in order to avoid microbial spoilage so that the beans can preserve their quality for long storage periods before processing [20]. The ash content of whole seeds slightly varied between samples, with values from 3.32 to 3.91% (dry basis (d.b.)), which agrees with values reported in the literature for red kidney beans [21]. The major compounds, starch and protein were measured separately in the coat (hull) and de-hulled seed (cotyledon) to obtain complimentary information about the location of these major compounds. Total starch content was only measured in cotyledons, since it is well known that seed coat does not possess starch [22]. Starch was found to be the main component in bean cotyledons, ranging from 41.59 to 48.47% (d.b.). The protein content ranged from 22.31 to 31.98% (d.b.) in the cotyledon and 4.61 to 6.24% (d.b.) in the seed coat. This is in line with the physiology of pulses, as protein is mainly found in the cotyledon as protein bodies [22]. All these values for starch and protein are in line with those previously reported in the literature [22,23,24,25,26]. In addition, the compositional results confirm that the growing location of the same bean genotype influences their composition, and, in turn, functionality during canning. 

The dry seed weight, seed coat to whole seed ratio (seed coat ratio), and WHC, are also reported in Table 2 and they were correlated with the major compounds. The weight per bean varied from 0.50 to 0.74 g, with only slightly higher values than those found in the literature amongst various types of *Phaseolus vulgaris* including red kidney beans [27,28]. The seed coat ratio ranged from 7.23 to 8.81%. It has been reported that seed coats constitute approximately 8% of the weight of the beans and that it, together with the total starch content in the cotyledon, influences the cooking time of the bean [29]. These weight and size differences can lead to different soaking and cooking behaviors of the seeds, as will be discussed below. As starch is the largest component in cotyledon (which constitutes the main fraction of the seed), the weight per bean significantly correlated to the starch content in the cotyledon (r = 0.912, *p* < 0.01) and, hence, it correlated negatively to protein content in cotyledon (r = −0.789, *p* < 0.01) and seed coat (r = −0.716, *p* < 0.01). 

The WHC of the beans, measured after soaking for 16 h at room temperature, ranged from 120.40 to 138.92% (expressed on weight percent basis). At room temperature, starch granules are insoluble in water and possess a low water absorption capacity, meaning that only protein and non-starch polysaccharides will be responsible for absorbing and bonding water [30]. In fact, WHC was positively correlated with the protein content in the cotyledon (r = 0.764, *p* < 0.01). In addition, WHC was negatively correlated to the weight per bean (r = −0.703, *p* < 0.01), which could be explained by the smaller surface area, and possibly relatively smaller micropyle and hilum sizes, also leading to higher seed coat ratio and protein content, as mentioned above.

### 3.2. Pasting Properties of the Whole Bean Flour

The results of RVA parameters obtained at high temperature conditions (130 °C) using water and brine as a cooking medium are found in Table 3 and Table 4, respectively. In addition, the differences in the average values of all samples for each pasting parameter are shown in Table 5. In Figure 1, pasting profiles obtained under pressurized high temperature (130 °C) conditions where compared to the standard 95 °C pasting profile using AACC 61-02.01 method [16]. During the conventional pasting profile at 95 °C, a defined peak viscosity was not clearly visible for the bean flour, i.e., the pasting curve was not fully developed. Therefore, the standard 95 °C pasting profile failed at providing quantitative rheological indicators (i.e., pasting parameters could not accurately be calculated). In contrast, the profiles obtained using the pressurized 130 °C procedure (with water or brine) displayed a complete pasting profile with pronounced peak, breakdown, and setback viscosities, as seen in Figure 1. Thus, only indicators from the 130 °C pasting profiles are discussed in this work. Pulse flours contain high amounts of protein and thick cell walls surrounding starch granules, which might restrict heat and mass transfer to the starch granules, impairing full gelatinization, which can be achieved with a conventional pasting profile only reaching 95 °C [15]. It can also be seen that, when using the cooking brine instead of ultrapure water as an RVA medium, peak time decreased, and peak viscosity increased significantly, as also displayed in Figure 1, indicating the faster hydration and cooking of bean flours in the presence of salts [29]. To understand the different pasting behavior observed for the two cooking media, two plausible explanations are possible. Firstly, increased denaturation temperature of the proteins vicilins (7S) and legumins (11S), which represent ~65% of all the common bean proteins, in the presence of ions in the medium during hydrothermal processing, was previously reported in the literature [31,32]. Increasing ionic strength impacts the functionality of the salt soluble vicilins (7S) and legumins (11S), leading to stronger bonds that make the protein more resistant to denaturation [33,34]. Therefore, the salt concentration in the brine used in this study would increase the denaturation temperature of the globulins up to 5 °C [32]. This would separate the state transitions between the 7S protein (with peak temperature of denaturation at ~78 °C in water [35]) and starch (with peak temperature of gelatinization at ~79 °C [36]), allowing the starch gelatinization to happen with less interruption from a denatured protein network. In fact, the presence of denatured proteins has been found to impair starch swelling and gelatinization [37,38]. These observations align with the results obtained by Joshi et al. [39], where the peak viscosity increased and peak time decreased with less protein in the mixture. Research conducted on the impact of salts on the pasting properties of isolated starches showed different results for different starch types [40,41]. However, no research has been conducted on the impact of salts on pulse flours, where a full pasting peak can only be obtained with high-pressure cooking conditions, as opposed to standard 95 °C procedures (Figure 1). Secondly, the change in the pasting curve shape may be due to the salt-solubility of globulins in the bean [42]. Up to ~90% of proteins in legumes are found to be salt-soluble [43]. As the brine solubilizes surface proteins bound to the starch granules and allows the starch to be more readily exposed to hydration [44], it would bring the earlier and more prominent pasting profile shown in Figure 1. In addition, the higher peak viscosity, breakdown and setback found with brine (pH 5.9) compared to water (pH ~7) also suggests a better solubilization of cell-wall components with slightly acidic conditions, which may be linked to the facilitation of the cleavage of strong bonds between protopectin and other cell wall materials [45].

As seen in Table 6 where Pearson correlations between bean attributes are summarized, starch and protein were significantly correlated to all RVA parameters except for peak time. In general, all pasting parameters that correlated positively with starch correlated negatively with protein in both seed coat and cotyledon fractions. Interestingly, using brine increased the correlation of all pasting parameters to the starch and protein content, except for trough viscosity.

The peak viscosity in brine was the parameter that correlated the strongest to total starch in cotyledon (r = 0.908, *p* < 0.01), meaning that a higher starch content (the one responsible for swelling) resulted in higher peak viscosity. Meanwhile, to reach half the viscosity value of the peak viscosity (time for PV/2) in both water and brine strongly correlated with protein in cotyledon and seed coat (r = 0.935 and r = 0.836, respectively, for water, and r = 0.928 and r = 0.884, respectively, for brine, all *p* < 0.01). The time for PV/2 is a more recent parameter that was adapted by Palabiyik et al. (2017) [46] to determine the resistivity of starch against processing conditions such as shearing, where a higher value means higher resistivity. Again, this will imply that a higher protein content results in higher resistivity of the flour particles to swell during heating, and, thus, a more delayed starch swelling and viscosity development.

Considering all the pasting parameters together, a higher amount of starch in the beans, and then, lower protein content, resulted in higher viscosity changes during the whole heating–cooling cycle, namely higher peak (maximum), trough (minimum) and final viscosities as well as higher setback and breakdown. In addition, lower pasting temperature and time to reach half the peak viscosity were also obtained with higher starch content and lower protein content. Altogether, these results would indicate that a higher and earlier swelling is obtained with the higher presence of starch in the cotyledon, as starch gelatinization and retrogradation are the main phenomena responsible for viscosity changes during the pasting profile. In contrast, a thicker or higher presence of a protein matrix in the bean flour, would hinder viscosity changes due to the physical limitations of starch swelling and water competition.

Among all pasting parameters, physical bean characteristics were best correlated (highest r coefficient values) to the time for PV/2 in both water and brine, indicative of the cotyledon resistivity to swell, demonstrating that time for PV/2 may be a good indicator for starch/protein content in beans. Together with time for PV/2, pasting temperature was also a good indicator of starch and protein content, as the high r values denote, regardless of the type of medium used, as there was no significant impact of the cooking medium on these values, as shown in Table 5. Another interesting point to note is that, although the peak viscosity changed in the curves shown in Figure 1, the time to reach half of this viscosity (Time for PV/2) did not, making it an interesting value to differentiate compositional attributes in bean flours. Time for PV/2 also showed a high correlation with WHC, seed weight and seed coat ratio (in brine, 0.804, −0.811 and 0.653, respectively, *p* < 0.01). These physical attributes were also correlated to other pasting parameters, like pasting temperature or final viscosity, as detailed in Table 6. It is important to highlight that these correlations, less strong than the protein and starch ones with pasting parameters, should be understood based on the existing correlation of physical and compositional attributes of the beans, as explained in Section 3.1. As an example, a higher WHC was obtained in beans with higher protein content in the sample, therefore, WHC is correlated in the same manner to pasting parameters as it does protein content.

### 3.3. Texture of the Canned Beans

**Figure 2:**
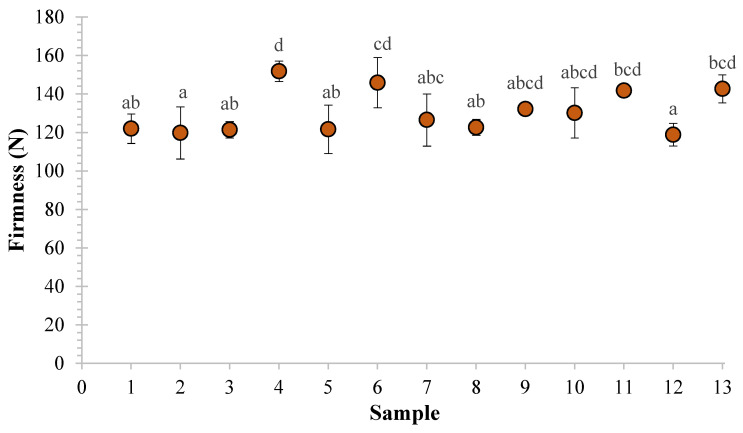
Visual of the distribution of average firmness values of bean samples. Error bars indicating standard deviation of firmness for each sample are included.

Firmness, obtained as the maximum-recorded force during textural analysis, was measured for the 13 bean samples 24 h after canning with cooking brine, and average values are reported in Figure 2. Canned beans had a firmness range of from 118.84 to 151.84 N with an average of 131.09 N.

Correlations between firmness values and all pasting parameters/bean characteristics are shown in Table 7. It was observed that the use of cooking brine in the RVA yielded stronger correlations with bean firmness than water. This event could be attributed to the enhancement of starch swelling by the presence of salts, as discussed in Section 3.2. Among all RVA parameters, the final viscosity (r = −0.951; *p* < 0.01) and trough viscosity (r = −0.926; *p* < 0.01), both using brine, had a very high negative correlation with the firmness of the canned beans. In addition, peak viscosity and setback were also negatively correlated to firmness (r = −0.820 and −0.741, respectively, *p* < 0.01). Although these results may seem contradictory, since a more enhanced viscosity profile with a higher final viscosity would be expected to result in higher firmness, these results should be explained based on compositional and structural attributes. Thus, the firmness of beans strongly correlated negatively with the starch (r = −0.768 *p* < 0.01) and positively with protein content (r = 0.785 *p* < 0.01) and seed coat ratio (r = 0.756 *p* < 0.01). It seems that smaller beans (i.e., higher coat to cotyledon ratio and lower weight per bean) with a high protein content would bring about a more compact cotyledon matrix that restricts starch from swelling and gelatinizing, which can be seen from a lower pasting profile with lower trough and final viscosity, resulting in canned beans with higher firmness. 

Although final viscosity alone had the highest correlation with bean firmness, analyzing the RVA parameters through stepwise linear regression (Table 8) led to models indicating that the highest amount of variance (adjusted R^2^ = 0.956) is covered when setback with brine is included in the model with either trough or final viscosity with brine (Model 2 and Model 4), rather than trough or final viscosity alone (Model 1 and Model 3). In this case, setback is a secondary RVA parameter and, therefore, it needs a primary indicator (either trough or final viscosity with brine) to set the base level of its impact for a correlation. Setback is reported to be an indicator of the short-term retrogradation ability of amylose after hydrothermal processing, and, thus, can be used to estimate the texture of a cooked starchy products [47,48,49]. In Table 8, it is shown that setback was able to distinguish more significant differences between samples (more subsets) compared to other parameters.

**Figure 3:**
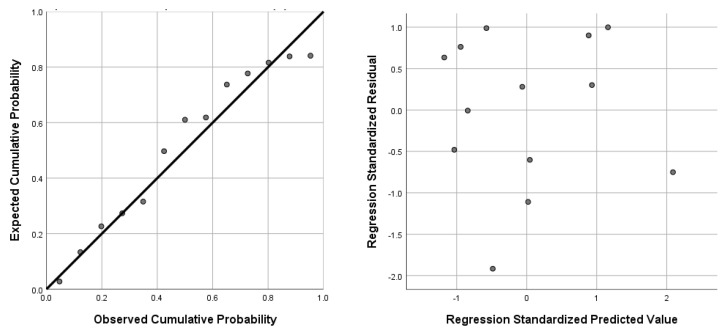
Normality plot of regression standardized residual and homoscedasticity scatter plot of models 2 (bean firmness with trough with brine and setback with brine) and 4 (bean firmness with final viscosity with brine and setback with brine).

As an example of the strength of the models, the normality and homoscedasticity of models 2 and 4 are shown in Figure 3. It is noteworthy that both models followed the exact same distribution of points. The variance inflation factors of model 2 (with trough viscosity and setback) and model 4 (with final viscosity and setback) were 1.323 and 4.990, respectively. As these values were less than 10, it means that there is absence of multicollinearity in these models. This confirms that the model meets all the assumptions required for linear regression, therefore the R-value of 0.981 can be applied.

## 4. Conclusions

The pressurized RVA 4800 at 130 °C was able to bring about a fully developed viscosity curve that provided rheological indicators that were strongly correlated to the instrumentally measured texture of canned beans. Furthermore, correlations became stronger when brine was used as a cooking medium in the RVA 4800, which was attributed to either or both the partial solubilization of proteins and cell wall polysaccharides and the increase in the denaturation temperature of proteins. Since the instrumental texture of beans is assessed on bean samples after the entire process of soaking, cooking and canning (which would take more than 16 h), the rapid determination of the pasting profile of the whole bean flour could save a significant amount of time (entire analysis in less than 30 min) during the prediction of the texture of canned beans. Future work should be conducted to extrapolate these correlations to other types of canned pulses. 

## Figures and Tables

**Table 1:** Standard pasting profile at 130 °C used for measuring pasting properties of whole bean flours (adapted from Perten Instruments, 2017).

Time (Minutes)	Parameter	130 °C Hold Temperature Cycle
00:00	Temperature	50 °C
00:00	Speed	960 rpm
00:10	Speed	160 rpm
01:00	Temperature	50 °C
07:40	Temperature	130 °C
10:10	Temperature	130 °C
16:50	Temperature	50 °C
18:50	End	

**Table 2:** Proximate analysis results of 13 red kidney bean samples.

Sample	Whole Bean Moisture (% w.b.)	Total Starch Cotyledon (%, d.b.)	Protein Cotyledon (%, d.b.)	Protein Seed Coat (%, d.b.)	Ash Whole Seed (%, d.b.)	Weight Per Bean (g)	Seed Coat Ratio (%)	WHC (%)
1	15.59cd ± 0.54	45.98cd ± 0.32	26.77j ± 0.01	4.91b ± 0.01	3.85ef ± 0.04	0.64de ± 0.02	7.42abc ± 0.15	130.78fg ± 0.11
2	14.58c ± 0.52	48.47e ± 0.38	22.36b ± 0.01	5.33g ± 0.01	3.40a ± 0.01	0.72fg ± 0.01	7.56abcd ± 0.15	123.89bc ± 0.57
3	12.64a ± 0.24	46.35cd ± 0.02	24.32f ± 0.01	4.90b ± 0.01	3.61bc ± 0.06	0.68ef ± 0.01	7.34ab ± 0.08	120.40a ± 0.62
4	14.45bc ± 0.01	41.59a ± 0.39	31.98m ± 0.00	6.24i ± 0.01	3.89f ± 0.01	0.50a ± 0.02	8.81f ± 0.04	133.06g ± 0.31
5	11.78a ± 0.77	46.58cd ± 0.34	22.79c ± 0.01	5.06d ± 0.01	3.78def ± 0.03	0.62cd ± 0.02	7.23a ± 0.02	127.42de ± 0.42
6	12.98ab ± 0.21	44.08b ± 0.11	30.12l ± 0.01	6.23hi ± 0.01	3.68bcd ± 0.03	0.58bc ± 0.02	7.89de ± 0.14	129.41ef ± 0.60
7	11.55a ± 0.26	45.63bc ± 0.12	25.04i ± 0.01	5.14e ± 0.01	3.84ef ± 0.01	0.69ef ± 0.01	7.33ab ± 0.07	121.16ab ± 1.29
8	14.31bc ± 0.71	47.68de ± 0.73	24.21e ± 0.01	5.30g ± 0.01	3.32a ± 0.03	0.71fg ± 0.01	7.66bcd ± 0.14	124.01bc ± 0.72
9	16.54d ± 0.26	46.91cde ± 1.14	24.95h ± 0.01	5.22f ± 0.00	3.73cde ± 0.04	0.74g ± 0.01	7.70cde ± 0.02	131.52fg ± 0.43
10	15.72cd ± 0.55	46.37cd ± 0.31	24.49g ± 0.01	4.61a ± 0.01	3.84ef ± 0.02	0.67ef ± 0.02	7.58bcd ± 0.15	128.94ef ± 0.30
11	16.86d ± 0.25	46.59cd ± 0.16	23.32d ± 0.00	5.03d ± 0.01	3.64bc ± 0.04	0.66de ± 0.01	7.74cde ± 0.07	123.77cd ± 2.09
12	16.41d ± 0.15	45.77cd ± 0.45	22.31a ± 0.01	4.97c ± 0.01	3.58b ± 0.01	0.64de ± 0.02	7.73cde ± 0.04	125.76cd ± 0.87
13	14.72c ± 0.23	41.95a ± 0.23	29.97k ± 0.01	6.20h ± 0.01	3.91f ± 0.04	0.55b ± 0.02	8.05e ± 0.12	138.92h ± 0.18

Average values ± standard deviation under the same column with same letter are not significantly different (*p* < 0.05). w.b.: wet basis, d.b.: dry basis, WHC: Water holding capacity.

**Table 3:** RVA parameters of 13 bean flours obtained using distilled water with a pressurized system reaching 130 °C.

Sample	Time for pv/2 (s)	Peak Viscosity (cp)	Trough Viscosity (cp)	Breakdown (cp)	Final Viscosity (cp)	Setback (cp)	Peak Time (s)	Pasting Temp. (°C)
1	285.40c ± 2.90	1628ef ± 72	998e ± 28	631g ± 61	1804ef ± 27	806def ± 7	7.38bc ± 0.22	83.27abc ± 0.45
2	264.53ab ± 0.37	1445bcde ± 1	945cde ± 6	500def ± 6	1685de ± 16	741bcd ± 22	6.97a ± 0.05	81.55a ± 1.70
3	272.22b ± 3.83	1178a ± 48	807ab ± 26	372abc ± 22	1489bc ± 38	683b ± 14	7.75d ± 0.04	82.68ab ± 0.03
4	300.59d ± 1.71	1001a ± 66	733a ± 28	268a ± 37	1249a ± 54	517a ± 26	7.20abc ± 0.13	85.38bcd ± 0.49
5	271.51ab ± 2.43	1420bcd ± 6	867bc ± 20	553efg ± 25	1609cd ± 19	742bcde ± 1	7.53cd ± 0.00	82.73ab ± 0.04
6	306.45d ± 0.52	1100a ± 43	799ab ± 9	301ab ± 52	1319a ± 6	520a ± 3	7.17ab ± 0.05	86.68d ± 1.10
7	263.19a ± 3.32	1380b ± 71	904cd ± 21	476cde ± 51	1712def ± 71	809f ± 50	7.40bc ± 0.10	81.95a ± 1.13
8	268.63ab ± 2.72	1391bc ± 10	973de ± 6	419bcd ± 16	1690de ± 19	717bc ± 13	7.07ab ± 0.09	82.70ab ± 0.07
9	270.02ab ± 1.19	1640f ± 43	1027e ± 12	612fg ± 36	1835f ± 33	808ef ± 24	7.15ab ± 0.04	81.63a ± 0.88
10	271.60ab ± 1.13	1538bcdef ± 83	949cde ± 43	589efg ± 54	1730def ± 54	781cdef ± 12	7.32bc ± 0.10	82.31a ± 0.84
11	265.82ab ± 5.72	1572cdef ± 33	988de ± 40	584efg ± 30	1833f ± 30	846f ± 11	7.17ab ± 0.05	81.53a ± 1.66
12	271.31ab ±0.11	1609def ± 23	994de ± 10	615fg ± 23	1832f ± 23	838f ± 13	7.10ab ± 0.04	82.65ab ± 0.00
13	307.40d ± 2.55	1087a ± 27	801ab ± 14	286a ± 27	1370ab ± 27	569a ± 13	6.87a ± 0.00	85.88cd ± 0.04

Average values ± standard deviation under the same column with same letter are not significantly different (*p* < 0.05). Time for PV/2: time to reach half peak viscosity.

**Table 4:** RVA parameters of 13 bean flours obtained using brine with a pressurized system reaching 130 °C.

Sample	Time for pv/2 (s)	Peak Viscosity (cp)	Trough Viscosity (cp)	Breakdown (cp)	Final Viscosity (cp)	Setback (cp)	Peak Time (s)	Pasting Temp. (°C)
1	291.70b ± 1.28	1928c ± 47	1006d ± 13	922cde ± 61	1849cde ± 12	843cde ± 25	6.57a ± 0.05	85.20bcd ± 0.07
2	282.56ab ± 2.07	1979c ± 28	989d ± 31	990de ± 3	1944e ± 35	955f ± 4	6.50a ± 0.14	82.68ab ± 0.04
3	285.54ab ± 2.49	1880c ± 16	980cd ± 5	901bcde ±11	1810cde ± 16	831bcde ± 11	6.57a ± 0.05	82.70ab ± 0.00
4	308.22c ± 2.16	1597ab ± 8	794a ± 8	803abc ± 16	1530a ± 7	736abc ± 16	6.64a ± 0.05	84.35abc ± 1.13
5	281.93ab ± 2.75	1949c ± 33	988d ± 23	961cde ± 11	1817cde ±57	829bcde ± 34	6.37a ± 0.05	82.75ab ± 0.07
6	308.16c ± 0.91	1605ab ± 19	881abc ± 15	724ab ± 4	1603ab ± 30	722ab ± 16	6.44a ± 0.05	85.90cd ± 0.07
7	286.46ab ± 1.17	1826bc ± 42	948cd ± 20	878bcde ± 22	1748bcd ± 23	800bcd ± 3	6.54a ± 0.09	84.35abc ± 1.13
8	279.51a ± 1.96	1895c ± 197	988d ± 55	907bcde ± 142	1896cde ± 128	908def ± 73	6.43a ± 0.14	83.15abc ± 0.64
9	283.23ab ± 3.59	1949c ± 40	923bcd ± 37	1026de ± 3	1802cde ± 45	879def ± 8	6.40a ± 0.10	82.78ab ± 0.04
10	279.24a ± 0.51	1795bc ± 28	944cd ± 7	851bcd ± 21	1749bcd ± 6	805bcd ± 1	6.47a ± 0.09	83.13abc ± 0.06
11	281.41a ± 5.32	1878c ± 40	835ab ± 43	1044e ± 4	1718bc ± 25	884def ± 18	6.44a ± 0.05	82.38a ± 1.73
12	279.13a ± 1.65	1913c ± 28	987d ± 13	926cde ± 41	1917de ± 33	931ef ± 46	6.37a ± 0.14	82.73ab ± 0.04
13	316.08c ± 2.31	1543a ± 25	919bcd ± 4	624a ± 21	1606ab ± 1	687a ± 4	6.60a ± 0.18	87.53d ± 0.04

Average values ± standard deviation under the same column with same letter are not significantly different (*p* < 0.05). Time for PV/2: time to reach half peak viscosity.

**Table 5:** Average values of RVA parameters at 130 °C for 13 bean flours using distilled water and brine.

Medium Type	Time for pv/2 (s)	Peak Viscosity (cp)	Trough Viscosity (cp)	Breakdown (cp)	Final Viscosity (cp)	Setback (cp)	Peak Time (s)	Pasting Temp. (°C)
Water	278.62a ± 16.30	1383a ± 214	907a ± 93	476a ± 129	1629a ± 200	722a ± 114	7.24a ± 0.25	83.15a ± 1.69
Brine	289.00a ± 13.83	1826b ± 149	937a ± 66	889b ± 118	1768b ± 127	831b ± 82	6.48b ± 0.09	83.81a ± 1.57

Average values ± standard deviation under the same column with same letters are not significantly different (*p* < 0.05). Time for PV/2: time to reach half peak viscosity.

**Table 6:** Pearson’s correlation between pasting properties of bean flours and compositional and physical properties of the dry beans studied.

RVA Parameter	RVA Medium	Whole Bean Moisture (%)	Total Starch in Cotyledon (%)	Protein in Cotyledon (%)	Protein in Seed Coat (%)	Ash (%)	Weight Per Bean (g)	Seed Coat Ratio (%)	WHC (%)
Time for PV/2 (s)	Water	0.209	−0.878 **	0.935 **	0.836 **	0.482	−0.869 **	0.702 **	0.841 **
	Brine	0.070	−0.888 **	0.928 **	0.884 **	0.461	−0.811 **	0.653 *	0.804 **
Peak viscosity (cP)	Water	0.226	0.716 **	−0.744 **	−0.786 **	−0.205	0.701 **	−0.525	−0.569 *
	Brine	−0.093	0.908 **	−0.888 **	−0.813 **	−0.495	0.816 **	−0.710 **	−0.703 **
Trough viscosity (cP)	Water	0.281	0.726 **	−0.686 **	−0.681 *	−0.354	0.755 **	−0.457	−0.586 *
	Brine	−0.332	0.616 *	−0.613 *	−0.585 *	−0.355	0.578 *	−0.780 **	−0.251
Breakdown (cP)	Water	0.175	0.671 *	−0.746 **	−0.820 **	−0.087	0.624 *	−0.547	−0.528
	Brine	0.067	0.799 **	−0.775 **	−0.697 **	−0.424	0.705 **	−0.46	−0.744 **
Final viscosity (cP)	Water	0.122	0.746 **	−0.787 **	−0.813 **	−0.270	0.768 **	−0.572 *	−0.665 *
	Brine	−0.062	0.867 **	−0.860 **	−0.717 **	−0.649 *	0.785 **	−0.688 **	−0.642 *
Setback (cP)	Water	−0.014	0.720 **	−0.824 **	−0.874 **	−0.187	0.734 **	−0.634 *	−0.691 **
	Brine	0.169	0.859 **	−0.851 **	−0.650 *	−0.728 **	0.762 **	−0.449	−0.801 **
Peak time (s)	Water	−0.517	0.225	−0.227	−0.547	0.217	0.166	−0.564 *	−0.225
	Brine	0.036	−0.574 *	0.629 *	0.391	0.420	−0.46	0.419	0.374
Pasting temp. (°C)	Water	0.084	−0.819 **	0.871 **	0.823 **	0.371	−0.828 **	0.574 *	0.772 **
	Brine	−0.020	−0.744 **	0.796 **	0.691 **	0.519	−0.627 *	0.362	0.788 **

** Correlation is significant at the 0.01 level (2-tailed). * Correlation is significant at the 0.05 level (2-tailed). WHC: Water-holding capacity, Time for PV/2: time to reach half peak viscosity.

**Table 7:** Correlations of textural properties of canned beans with the pasting properties at high temperature (130 °C) and compositional and physical measurements of dry seeds.

Parameter		Firmness of Beans (N)
Time for PV/2 (s)	Water	0.704 **
	Brine	0.691 **
Peak viscosity (cP)	Water	−0.583 *
	Brine	−0.820 **
Trough viscosity (cP)	Water	−0.572 *
	Brine	−0.926 **
Breakdown (cP)	Water	−0.560 *
	Brine	−0.516
Final viscosity (cP)	Water	−0.631 *
	Brine	−0.951 **
Setback (cP)	Water	−0.644 *
	Brine	−0.741 **
Peak time (s)	Water	−0.337
	Brine	0.321
Pasting temp (°C)	Water	0.634 *
	Brine	0.499
Total Starch in cotyledon (%)		−0.768 **
Protein in cotyledon (%)		0.785 **
Protein in seed coat (%)		0.748 **
Ash (%)		0.500
Whole bean moisture (%)		0.205
Weight per bean (g)		−0.730 **
Seed coat ratio (%)		0.756 **
WHC (%)		0.557 *

** Correlation is significant at the 0.01 level (2-tailed). * Correlation is significant at the 0.05 level (2-tailed).

**Table 8:** Linear Regression of Firmness of Beans with RVA variables.

Model ^a^	R	R^2^	Adjusted R^2^	Standard. Error of the Model (%)
1	0.926	0.857	0.844	4.34
2	0.981	0.963	0.956	2.30
3	0.951	0.904	0.895	3.55
4	0.981	0.963	0.956	2.30

^a^ Dependent Variable: Firmness of Beans. Model 1 Predictors: (Constant), Trough viscosity with Brine; Model 2 Predictors: (Constant), Trough viscosity with Brine, Setback with Brine; Model 3 Predictors: (Constant), Final viscosity with Brine; Model 4 Predictors: (Constant), Final viscosity with Brine, Setback with Brine.
